# Distinct photo-oxidation-induced cell death pathways lead to selective killing of human breast cancer cells

**DOI:** 10.1038/s41419-020-03275-2

**Published:** 2020-12-14

**Authors:** Ancély F. Dos Santos, Alex Inague, Gabriel S. Arini, Letícia F. Terra, Rosangela A. M. Wailemann, André C. Pimentel, Marcos Y. Yoshinaga, Ricardo R. Silva, Divinomar Severino, Daria Raquel Q. de Almeida, Vinícius M. Gomes, Alexandre Bruni-Cardoso, Walter R. Terra, Sayuri Miyamoto, Maurício S. Baptista, Leticia Labriola

**Affiliations:** 1 Departamento de Bioquímica, Instituto de Química, Universidade de São Paulo (USP), São Paulo, 05508-000 Brazil; 2 Faculdade de Ciências Farmacêuticas de Ribeirão Preto, Universidade de São Paulo (USP), Ribeirão Preto, 14040-903 Brazil

**Keywords:** Lipidomics, Necroptosis, Stress signalling, Breast cancer

## Abstract

Lack of effective treatments for aggressive breast cancer is still a major global health problem. We have previously reported that photodynamic therapy using methylene blue as photosensitizer (MB-PDT) massively kills metastatic human breast cancer, marginally affecting healthy cells. In this study, we aimed to unveil the molecular mechanisms behind MB-PDT effectiveness and specificity towards tumor cells. Through lipidomics and biochemical approaches, we demonstrated that MB-PDT efficiency and specificity rely on polyunsaturated fatty acid-enriched membranes and on the better capacity to deal with photo-oxidative damage displayed by non-tumorigenic cells. We found out that, in tumorigenic cells, lysosome membrane permeabilization is accompanied by ferroptosis and/or necroptosis. Our results also pointed at a cross-talk between lysosome-dependent cell death (LDCD) and necroptosis induction after photo-oxidation, and contributed to broaden the understanding of MB-PDT-induced mechanisms and specificity in breast cancer cells. Therefore, we demonstrated that efficient approaches could be designed on the basis of lipid composition and metabolic features for hard-to-treat cancers. The results further reinforce MB-PDT as a therapeutic strategy for highly aggressive human breast cancer cells.

## Introduction

Breast cancer is the most frequent malignancy in women worldwide^1,2^. In its advanced stages, when distant organ metastases occur, it is considered incurable with the currently available therapies^2^. The reason being that metastatic lesions are usually multiple, molecular and cellular heterogeneous, and resistant to conventional treatments^3,4^. Thus, effective and safe therapies for this stage of the disease are still needed.

Photodynamic therapy (PDT) has been the focus of several cancer centers, as it might represent an important advancement in treatment due to its high but also controlled cytotoxic effect^4–6^. In addition, the enhanced antitumor effects combining PDT and chemotherapies have already been demonstrated in preclinical studies on breast cancer^3,4,7^. As PDT was highly effective in eliminating microscopic disease in the post-surgical tumor bed and preventing a secondary disease in mice^8,9^, it appears as a promissory therapeutic alternative for high recurrence types of cancer. PDT consists in the uptake of a photosensitizer (PS) molecule, which, upon excitation by light in a determined wavelength, reacts with oxygen and generates oxidant species (radicals, singlet oxygen, and triplet species) in target tissues, leading to photo-oxidative stress (PhOxS)^10,11^, which results in photodamage of membranes and organelles^12,13^. The extent of the damage and the cell death mechanisms involved are dependent on the PS type, concentration, subcellular localization, as well as the amount of energy and fluence rate applied, and also on the intrinsic characteristics of each tumor type^14–17^. The bottleneck of PDT is that little is known about the complex molecular mechanisms behind its cytotoxicity and even less about the factors that could improve its specificity against aggressive cancer cells^4,7,18^. To address these underpinnings, our group has been studying PDT using methylene blue as PS (MB-PDT) in human breast cell (BC) models.

In previous studies, we have reported that there were differences in MB-PDT sensitivity regarding MB concentration, time to achieve maximal cell death, and the effect of fluence rate^14,19^. Moreover, our results have shown that non-tumorigenic BCs are more resistant to MB-PDT, whereas the very aggressive triple-negative breast cancer cells (TNBCs) displayed the highest susceptibility^19^. However, the mechanisms behind these effects are still not well understood. Given the relevance of oxidative processes to cell death, in the present study, we set out to unveil the molecular mechanisms triggered by this PhOxS therapy, which are responsible for its selectivity in the elimination of cancer cells and its potential as a future breast cancer adjuvant treatment.

## Results

### Human BCs presenting variations in PDT sensitivity displayed differential cellular lipid composition

**Fig. 1:**
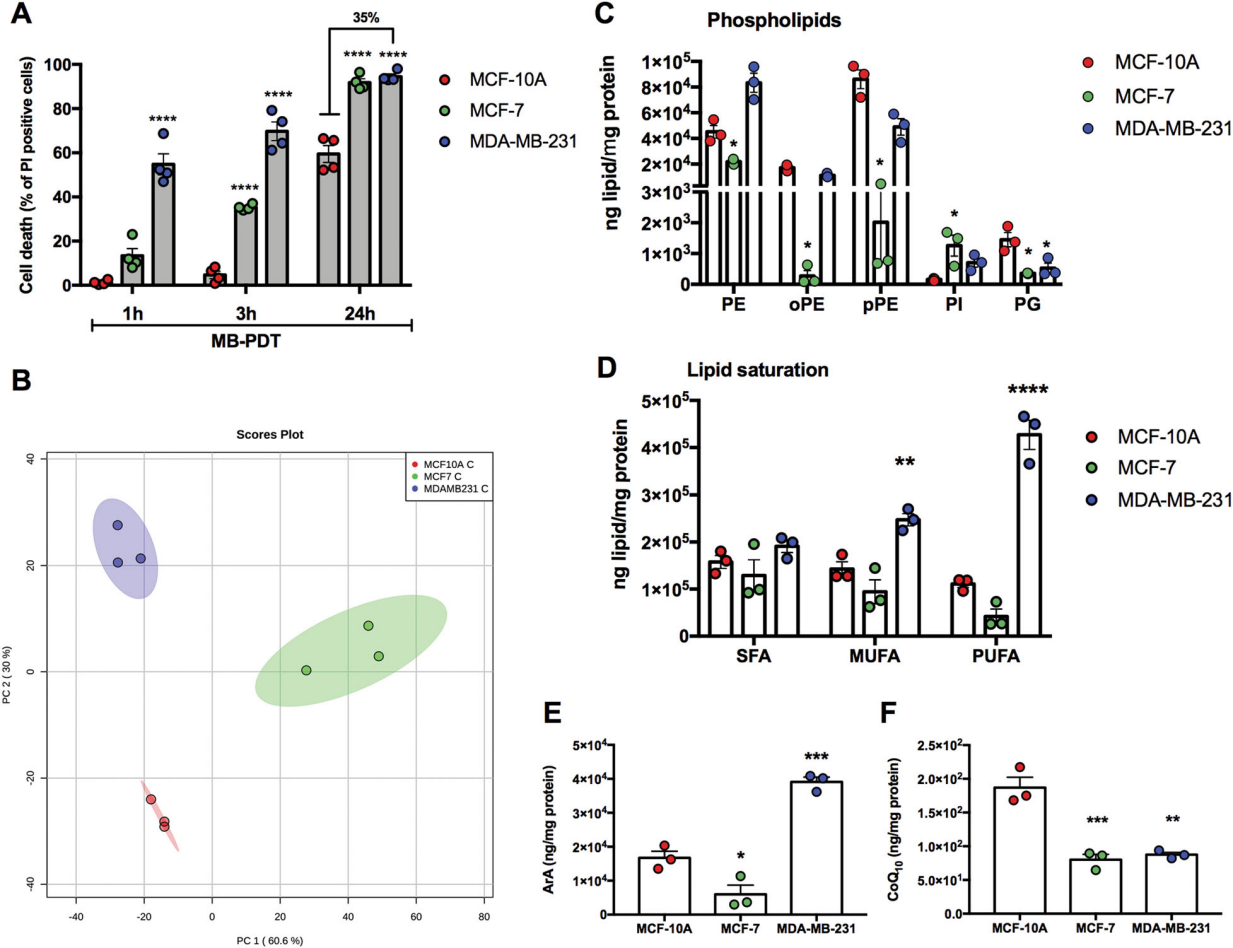
Human breast cells display differential sensitivity to MB-PDT and lipid composition. **A** Cell death induction after 1, 3, or 24 h of MB-PDT treatment. **B** Score plot of the principal component analysis (PCA) of lipidomic data of BC. **C** Abundance of the glicerophospholipids: PE, oPE, pPE, PI and PG. **D** Abundance of lipids grouped by the degree of fatty acid chain saturation. Saturated fatty acid (SFA), monounsaturated fatty acid (MUFA), and polyunsaturated fatty acid (PUFA). **E** Abundance of ArA-containing lipids in BC. **F** Abundance of CoQ_10_. Dot color representation: MCF-10A in red, MCF-7 in green, MDA-MB-231 in blue. ****p < 0.0001, ***p < 0.001, **p < 0.005, *p < 0.05 vs. MCF-10A. Results are presented as mean ± SEM. Each dot represents an independent experiment. *n* ≥ 3 independent experiments.

We first confirmed our previous results^19^ by showing that cell death after MB-PDT exerted a higher impact in the malignant cell lines, the TNBC cells (MDA-MB-231) being the most susceptible (Fig. 1A). As MB-PDT relies on a massive intracellular generation of oxidant species^14,19,20^, with a widespread impact in membranes, we then evaluated whether there was a link between the cellular lipid profile and the sensitivity to MB-PDT. By performing comparative lipidomics profiling, we identified and grouped around 487 different species as sphingolipids, glycerophospholipids (GPs), neutral lipids (NLs), free fatty acids, and coenzyme Q (CoQ). The two first components of the principal component analysis (PCA) explained 90.6% of the lipid content variance and indicate a clear clustering between BC types (Fig. 1B). Among all the obtained lipid classes, the main differences between BC were found on the amount of some NL (cholesteryl ester, ceramide, diacylglycerol, and triacylglycerol), CoQ_10_, and some species of GP [phosphatidylethanolamine (PE), phosphatidylinositol (PI), phosphatidylglycerol, and phosphatidylcholine, besides plasmanyl (o)- and plasmenyl (p)-GP] (Fig. 1C and Supplementary Fig. S1A, B). NL species were more abundant in breast cancer cells (Supplementary Fig. S1A). A higher proportion of PI was found in malignant cells but in this case mainly in MCF-7 cells, a model of the less aggressive luminal A subtype (Fig. 1C). MDA-MB-231, a TNBC cell type, and MCF-10A, non-tumorigenic BCs, displayed similar levels of PE; the lowest levels of this class of lipids were observed in MCF-7 cells (Fig. 1C). Considering all lipid species, MDA-MB-231 presented the highest levels of monounsaturated and polyunsaturated fatty acids (PUFAs) (Fig. 1D), with special attention to arachidonic (ArA) and docosatetraenoic/adrenic acid (AdrA) PUFAs (Fig. 1E and Supplementary Fig. S1C). MDA-MB-231 cells also presented the highest abundance of these acids esterified with PE (Supplementary Fig. S1D, E). Non-tumorigenic cells displayed higher levels of CoQ_10_, compared to malignant cells (Fig. 1F).

### MB-PDT induces ferroptosis in TNBC cells

The previous observations led us to hypothesize whether MB-PDT could induce ferroptosis, a form of regulated cell death (RCD), which is initiated by oxidative insults that occurs mainly towards PUFA, as ARA or AdrA^21^ (especially if they are esterified in PE^22^), an iron available as labile iron pools (LIPs).

**Fig. 2:**
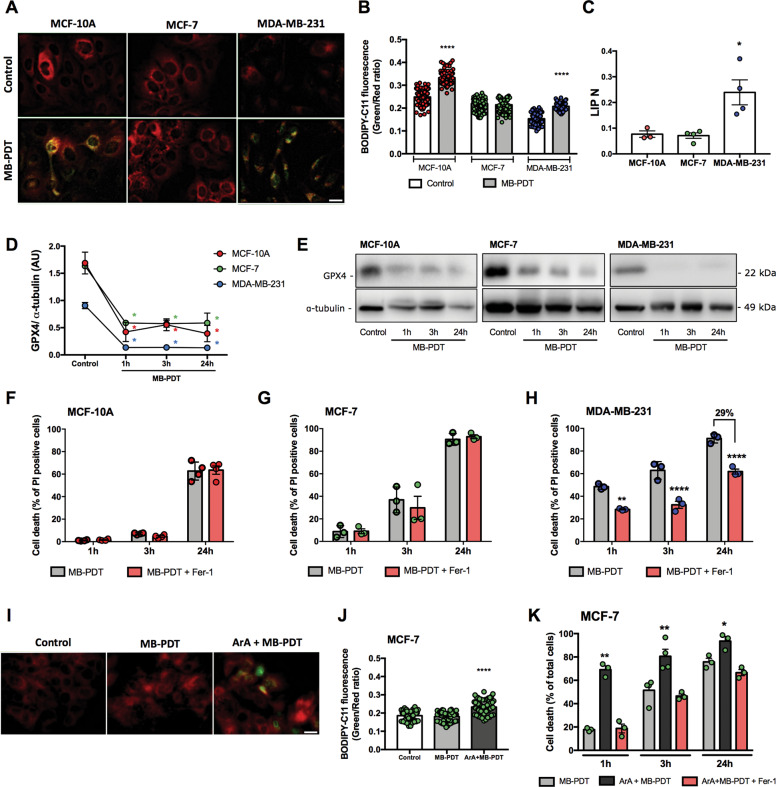
MB-PDT induces ferroptosis in PUFA- and LIP-enriched cells. **A** Representative images of lipid peroxidation in cells submitted or not to MB-PDT. Reduced (red) or oxidized (green) BODIPY-C11. **B** Graphical representation of the quantification of the oxidized/reduced BODIPY-C11 ratio/cell. Each dot represents an individual cell. A minimum of 100 cells was analyzed/experiment. ****p < 0.0001 vs. control of each cell line. **C** Intracellular labile iron poll (LIP), normalized by the total intracellular calcein incorporated per cell (LIPN). *p < 0.05 vs. MCF-10A. **D** GPX4 protein quantification in cells after 1, 3, and 24 h of being submitted or not to MB-PDT (Control). *p < 0.05 vs. control of each cell line. **E** Representative images of western blottings of GPX4 of BC after being treated or not with MB-PDT, as indicated. Middle panels show the percentage of cell death after MB-PDT (1, 3, or 24 h) in cells pretreated or not with Fer-1: **F** MCF-10A, **G** MCF-7, and **H** MDA-MB-231. **p < 0.005 vs. MB-PDT; ****p < 0.0001 vs. MB-PDT. **I** Representative images of lipid peroxidation in MCF-7 cells pre-incubated or not with ArA before being submitted or not to MB-PDT. **J** Corresponding quantification (as described in item **B**) of oxidized/reduced BODIPY-C11 ratio in MCF-7 cells. ****p < 0.0001 vs. control. **K** Cell death percentage of MCF-7 cells pretreated or not with Fer-1 and/or ArA as indicated after 1, 3, or 24 h of photo-oxidation induction. **p < 0.005 vs. MB-PDT; *p < 0.05 vs. MB-PDT. *n* ≥ 3 independent experiments. Dot color representation: MCF-10A in red, MCF-7 in green, and MDA-MB-231 in blue. Results are presented as mean ± SEM.

The presence of the key components of ferroptosis ACSL4 (acyl-CoA synthetase long-chain family member 4) and GPX4 (gluthatione peroxidase 4) were evaluated. Unlike MCF-7 cells, MCF-10A and MDA-MB-231 expressed ACSL4 (Supplementary Fig. 2). Non-tumorigenic cells presented higher levels of GPX4, compared to the other cell lines (Supplementary Fig. 2). Lipid peroxidation after MB-PDT treatment was only increased in MCF-10A and MDA-MB-231 cells (Fig. 2A, B). In addition, MDA-MB-231 displayed the highest basal levels of LIP (Fig. 2C).

To investigate the role of ferroptosis after photo-oxidation, cells were pretreated with the ferroptosis inhibitor ferrostatin-1 (Fer-1), followed by MB-PDT. Results showed that despite the fact that MB-PDT promoted depletion of GPX4 in all cell lines tested (Fig. 2D, E), only MDA-MB-231 cells were cytoprotected by Fer-1 (Fig. 2F–H). These results indicated that MDA-MB-231 cells were not capable of coping with lipid peroxidation and consequently more susceptible to ferroptosis.

To test whether PUFAs were required to induce ferroptosis and hence increased MB-PDT cytotoxicity, MCF-7 cells were pre-incubated with ArA. After MB-PDT, the cells underwent not only lipid peroxidation (Fig. 2I, J) but became more sensitive to photo-oxidation (Fig. 2K). MB-PDT was now able to induce ferroptosis, as Fer-1 significantly inhibited cell death (Fig. 2K). These data demonstrated that lipid peroxidation was a cytotoxic insult triggered by MB-PDT and cells presenting low abundance of PUFAs were less affected. In addition, they indicated high levels of PUFA are required to undergo ferroptosis in response to MB-PDT.

### Non-tumorigenic cells are more prone to mount an efficient antioxidant response against MB-PDT

**Fig. 3:**
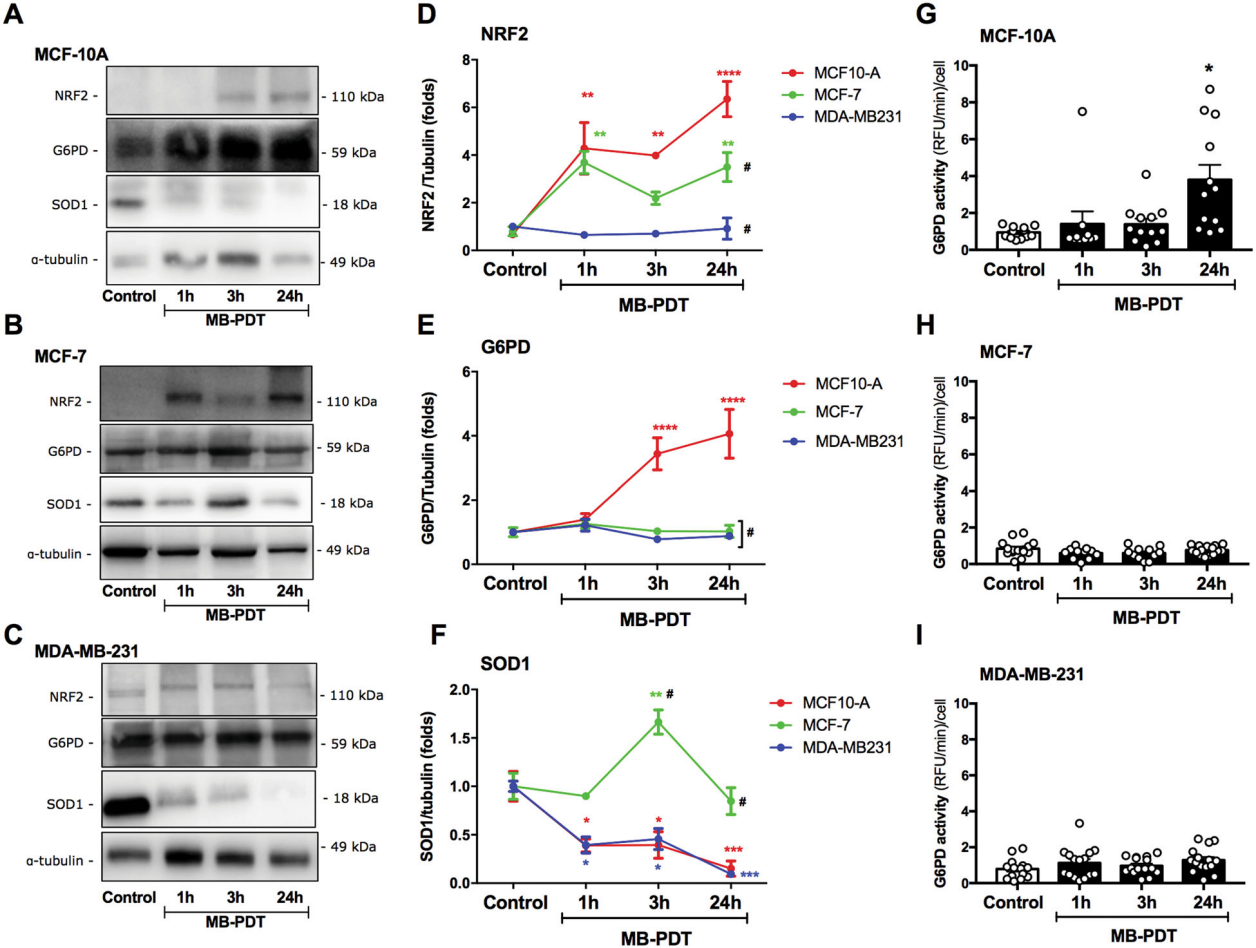
Non-tumorigenic breast cells display antioxidant response to MB. Representative images of western blottings of NRF2, G6PD, and SOD1 for each BC after being submitted or not with MB-PDT: **A** MCF-10A, **B** MCF-7, and **C** MDA-MB-231. Western blotting quantifications of **D** NRF2, **E** G6PD, and **F** SOD1 of each BC treated or not with MB-PDT (PDT). Results are presented as folds vs. Control condition. Color representation: MCF-10A in red, MCF-7 in green, and MDA-MB-231 in blue. G6PD activity in **G** MCF-10A, **H** MCF-7, and **I** MDA-MB-231 cells after being submitted or not to MB-PDT (PDT). *n* ≥ 3 independent experiments. ****p < 0.0001, ***p < 0.001, **p < 0.005, *p < 0.05 vs. Control; #*p* < 0.05 vs. MCF-10A at each time point. Results are presented as mean ± SEM.

To understand the resistance of MB-PDT-induced cell death, basal levels of antioxidant-related proteins were analyzed. MCF-7 cells displayed the highest basal protein levels of glucose 6-phosphate dehidrogenase (G6PD), copper/zinc, and manganese superoxide dismutases (SOD1 and SOD2) (Supplementary Fig. 3A–F). MCF-10A cells presented the highest expression of glutathione (GSH) synthetase when compared to the extremely low values found in tumorigenic cells (Supplementary Fig. 3A, G), indicating that these breast cancer cells possess less capacity to de novo GSH synthesis, and that TNBC cells would be potentially more susceptible to oxidative damage^19^. Indeed, MB-PDT induced a significant depletion of reduced GSH in tumorigenic cells (Supplementary Fig. 2D). To explore deeper the antioxidant response to MB-PDT, we analyzed the levels of enzymes involved in this process before and after photo-oxidation. Despite the lack of differences in basal NF-E2-related factor 2 (NRF2)’s levels between cells (Supplementary Fig. 3A, E), upon MB-PDT this transcription factor was significantly increased in MCF-10A, slightly increased in MCF-7, and not modulated in MDA-MB-231 cells (Fig. 3A–C, D). NRF2 rise was accompanied by higher G6PD levels (Fig. 3A–C, E) and activity (Fig. 3G–I) only in the non-tumorigenic cells. Although cellular levels of SOD1 significantly peaked at 3 h after MB-PDT and remained high up to the last time point in MCF-7, a pronounced depletion of was observed early in both MCF-10A and MDA-MB-231 cells (Fig. 3A–C, F) after treatment.

These results allowed us to conclude that non-tumorigenic BCs were able to activate a proficient antioxidant response through the increase of G6PD, which in turn would lead to the higher production of NADPH that could then be used to regenerate GSH, as well as reduced CoQ_10_, contributing to the detoxification process and thus inhibiting cell death.

### MB-PDT can also trigger necroptosis

**Fig. 4:**
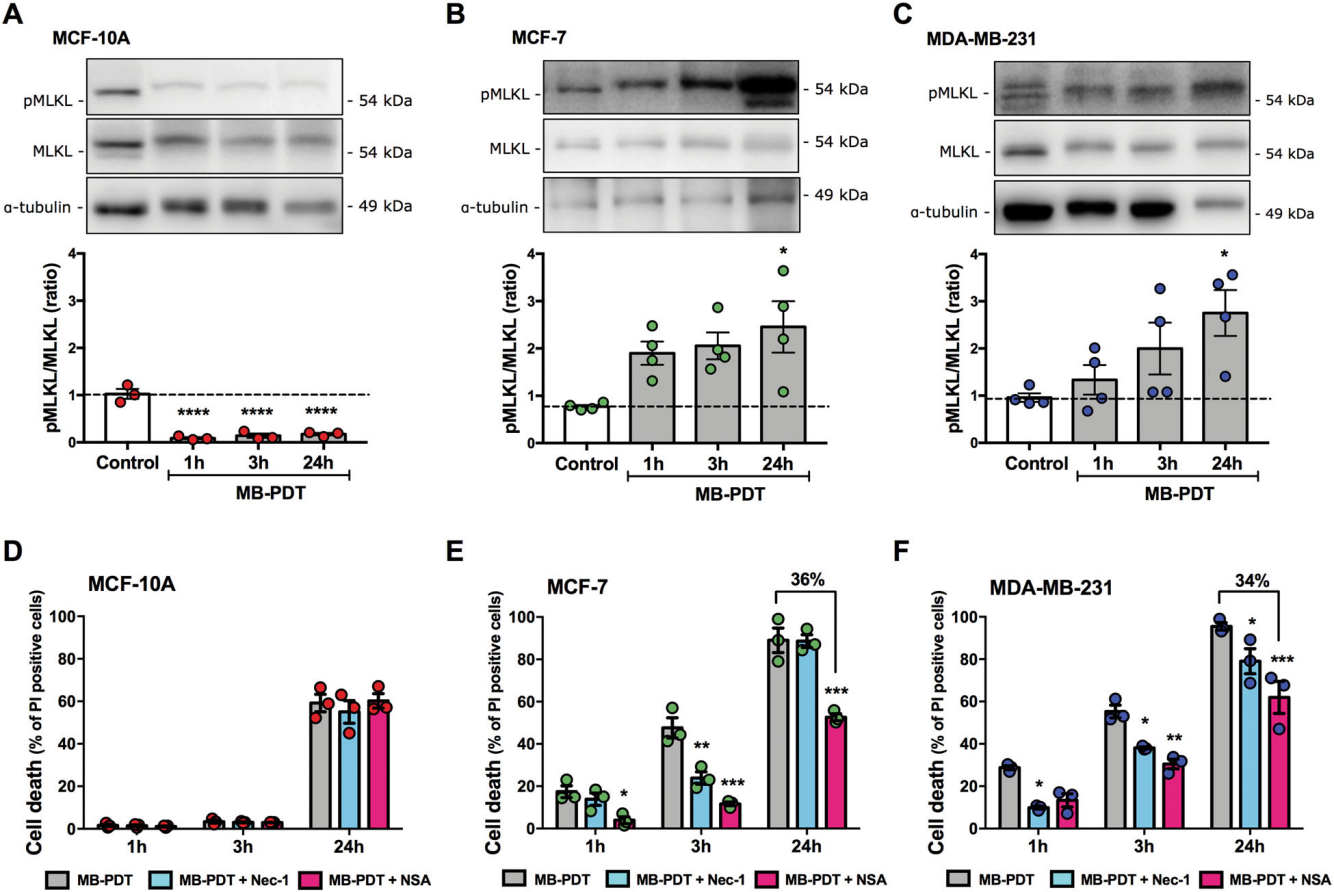
MB-PDT activates necroptosis in tumorigenic breast cells. **A** MCF-10A, **B** MCF-7, and **C** MDA-MB-231 cells were submitted or not to MB-PDT. One, 3, and 24 h after irradiation, cells were lysed and protein extracts were submitted to western blotting. Representative images of western blottings of pMLKL, MLKL, and tubulin, and the corresponding quantification of pMLKL/MLKL ratio are shown. *p < 0.05 vs. Control (*n* = 4 independent experiments). **D** MCF-10A, **E** MCF-7, and **F** MDA-MB-231 were pretreated or not with Nec-1 or NSA, and then submitted or not to MB-PDT. Cell death was evaluated 1, 3, or 24 h after irradiation. ***p < 0.001, **p < 0.005, *p < 0.05 vs. MB-PDT. Dot colors representation: MCF-10A in red, MCF-7 in green, and MDA-MB-231 in blue. Results are presented as mean ± SEM (*n* = 3 independent experiments).

As PDT can also elicit other RCD pathways, basal levels of key components of necroptosis, such as RIPK1, RIPK3, and MLKL (receptor interacting protein kinases-1 and -3, and mixed lineage kinase domain-like protein, respectively), were checked. MCF-7 cells displayed the highest levels of these proteins (Supplementary Fig. 4A–D). Activation of necroptosis was next assessed by monitoring MLKL phosphorylation (pMLKL). pMLKL levels increased only in tumorigenic cells submitted to MB-PDT (Fig. 4A–C). The role of necroptosis in MD-PDT cell death was further investigated with necrostatin-1 (Nec-1) or necrosulfonamide (NSA) pretreatments and by silencing the expression of RIPK3 or MLKL. Only tumorigenic cells were protected from MB-PDT effects in the presence of the necroptosis inhibitors (Fig. 2D–F and Supplementary Fig. 4E–H). Altogether, these data indicated that MB-PDT was capable of activating necroptosis only in the tumorigenic cells.

### MB-PDT induces lysosome damage

**Fig. 5:**
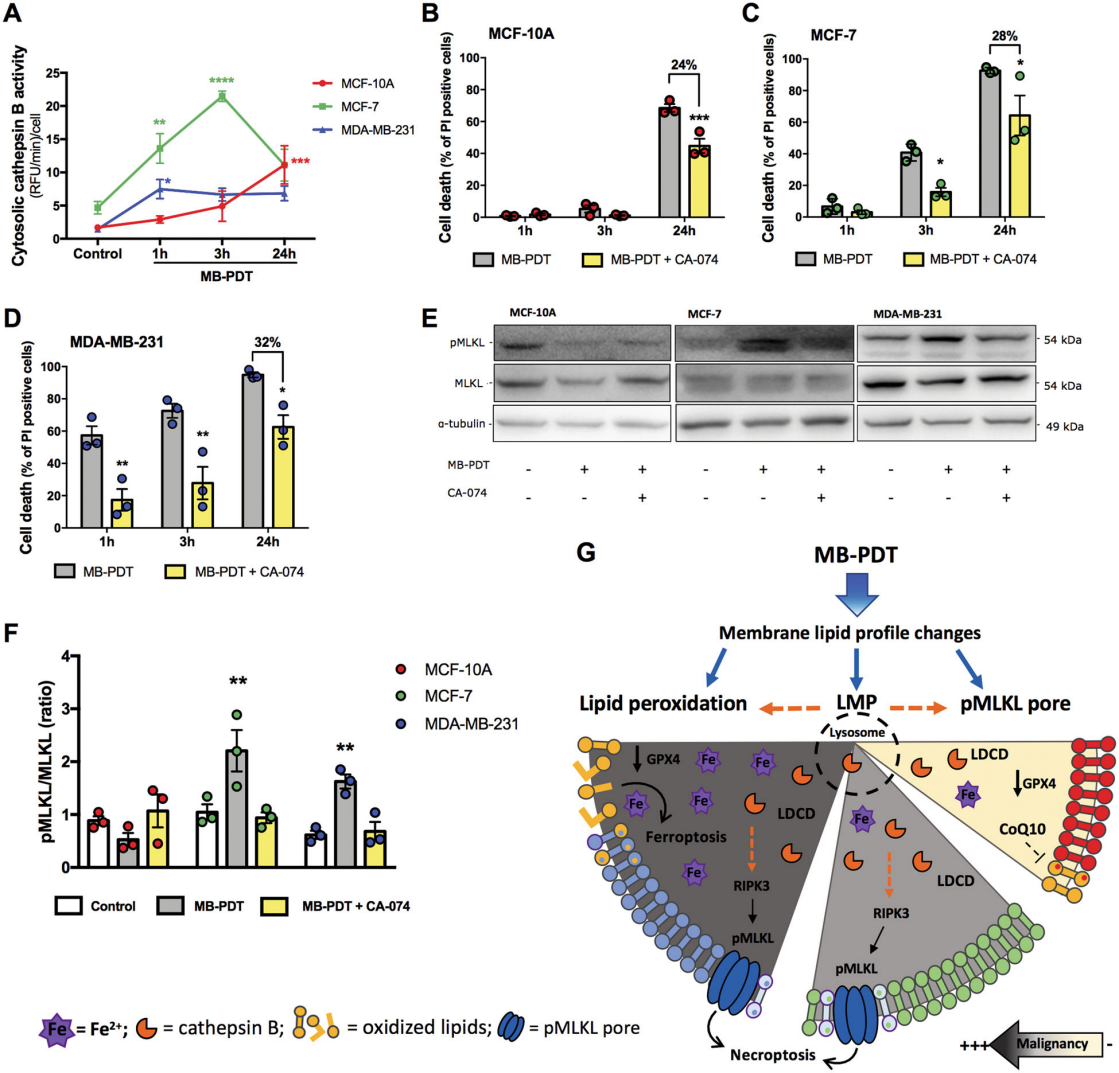
MB-PDT-induced LMP is involved on cell death of breast cells. **A** Cells were submitted or not to MB-PDT and cytosolic cathepsin B activity was analyzed after 1, 3, or 24 h of cell treatment. ****p < 0.0001, ***p < 0.001, **p < 0.005, *p < 0.05 vs. Control. **B** MCF-10A, **C** MCF-7, and **D** MDA-MB-231 cells were pretreated or not with CA-074 and then submitted or not to MB-PDT. Cell death was analyzed after 1, 3, 24 h of cell irradiation: ***p < 0.001, **p < 0.005, *p < 0.05 vs. MB-PDT. **E** Representative images of western blottings of pMLKL, MLKL, and tubulin of BC treated or not with CA-074 and MB-PDT after 24 h, as indicated. **F** pMLKL/MLKL ratio of cells after 24 h they were treated or not with CA-074 and then submitted to MB-PDT. **p < 0.005 vs. Control. Dot colors representation: MCF-10A in red, MCF-7 in green, and MDA-MB-231 in blue. *n* ≥ 3 independent experiments. Graph results are presented as mean ± SEM. **G** Cell death mechanisms activated by MB-PDT in BC ranging from non-malignant to very aggressive tumorigenic cells (from right to left). Upper part shows that photo-oxidation induces membrane lipid profile changes such as lipid peroxidation, LMP, and/or pMLKL pore formation. Bottom part of the figure represents the differential lipid composition of each BC analyzed (represented by different lipid membrane colors) and which mechanisms are activated in each one (LDCD: lysosome-dependent cell death, necroptosis, or ferroptosis). MDA-MB-231 cells: dark gray background, with higher proportion of lipids to undergo lipid peroxidation, MCF-7: gray background displaying lipids not susceptible to peroxidation, MCF-10A: yellow background and bearing intermediate abundance of lipids being susceptible to oxidation.

We have previously reported lysosomal accumulation of MB in BC^19^. We now hypothesize whether photoactive MB could damage lysosome membrane and thus induce lysosome-dependent cell death (LDCD). In fact, MB-PDT induced lysosomal membrane permeabilization (LMP), as we detected increased cathepsin B activity in the cytosol of cells after photoxidation (Fig. 5A). LDCD’s involvement was then shown by the reduction of MB-PDT cytotoxicity observed when cathepsin B was inhibited with the small molecule CA-074 (Fig. 5B–D). These results indicated that LMP was a common event triggered by MB-PDT in all cells analyzed. In addition, as the levels of pMLKL were decreased upon cathepsin B inhibition, the results pointed at a cross-talk between LDCD and necroptosis induction after photo-oxidation (Fig. 5E, F).

Altogether, we demonstrated that MB-PDT trigger multiple RCD in BC by inducing modifications in lipid membranes. These molecular mechanisms leading to cell death induction were both cell specific and PS dependent (Supplementary Figs. 5 and 6). Indeed, when BC were submitted to Hypericin-PDT different cell death pathways were activated (Supplementary Fig. 6). MCF-7, which lack capase-3^23^, became resistant to Hypericin-PDT, whereas non-tumorigenic cells died in a proportion similar to the one observed in TNBC cells, supressing the efficiency and selectivity displayed by MB-PDT.

In sum, in malignant cells, our data pointed that upon MB-PDT, LMP was followed by pMLKL and cell death. Cells containing high amounts of PUFAs were also able to undergo ferroptosis triggered via lipid peroxidation, GPX4 depletion and failure to activate responses involved in oxidative damage detoxification (Fig. 5G).

## Discussion

**Fig. 6:**
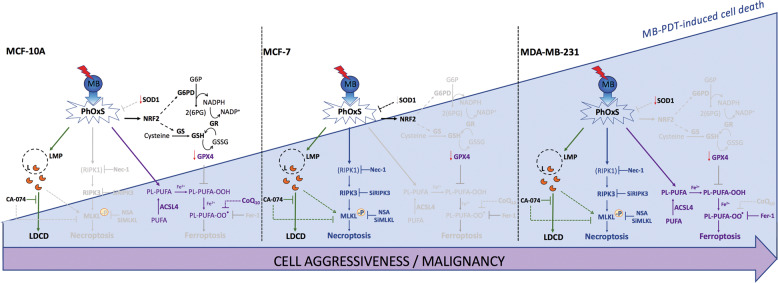
Scheme summarizing the antioxidant and cell death mechanisms activated in human breast cells by MB-PDT. Breast cells display different susceptibility to photo-oxidative stress (PhOxS) induced by MB-PDT, being the highest effect observed in MDA-MB-231. In this cell line, no antioxidant response was mounted upon PhOxS. In addition, low levels of GPX4 and CoQ_10_, combined with high amount of iron (Fe^2+^) and PUFA-phospholipid content (PL-PUFA), resulted in ferroptosis activation by MB-PDT (purple arrows and letters). This cell death was inhibited by Fer-1 pretreatment. Lysosomal damage was observed in all cell lines, evidenced by the release of cathepsin B through lysosomal membrane permeabilization (LMP) (green arrows and letters). Pretreatment with CA-074, a cathepsin B inhibitor, alleviated cell death. In both tumorigenic cells, MDA-MB-231 and MCF-7, necroptosis activation with MLKL plasma membrane pore formation (blue arrows and letters) was observed. Inhibition of RIPK1, RIPK3, or MLKL phosphorylation, by gene silencing or pretreatment with Nec-1 or NSA, rescue tumorigenic cells from death. A possible link between LMP and necroptosis was found in tumorigenic cells (green dotted arrows). As MCF-7 cells lack significant amounts of oxidizable phospholipids, lipid peroxidation was not observed and, therefore, ferroptosis did not contribute to death. However, a complete antioxidant response was not sustained in these cells, making them also highly affected by MB-PDT. The scenario after PhOxS was quite different for MCF-10A cells. Even undergoing LMP and lipid peroxidation, they were significantly more resistant to MB-PDT than the other cells. Neither ferroptosis nor MLKL phosphorylation nor necroptosis were observed.

In this study, we went further in addressing a possible association of a defined lipid profile with aggressiveness and susceptibility to undergo different cell death subroutines upon massive oxidant species generation^24,25^. Our data have reinforced the potential of this therapy to present fewer side effects in non-cancerous breast tissue, by providing several evidences on how PhOxS triggered by MB-PDT has barely affected antioxidant capacity of non-tumorigenic cells to deal with oxidative damage (Fig. 6).

Although decades of research efforts have produced thousands of publications in the field of PDT, few authors have reported on the factors that affect efficiency and selectivity of a PS towards a certain type of disease. We accept this challenge by studying the cellular makeups in lipid composition, antioxidant machinery, and cell death mechanisms induced by MB-PDT in breast cancer cells (Fig. 6).

Increasing amount of evidences have highlighted the role of lipids as triggers, executors, or modulators of plasma membrane components that act as platforms for RCD execution^26–28^. Moreover, lipids displaying different susceptibilities to undergo chemical modifications can be involved on cell death induction by modulating membrane physicochemical properties^29,30^. For example, exposure of lipids to sources of free radicals, molecular oxygen, and redox-active metal ions results in lipid peroxidation^31^. In the context of PDT, the direct contact within lipid membranes and the excited triplet state of the PS, during photosensitized oxidations, also results in membrane peroxidation^13^. This study provided strong evidences of membrane lipid peroxidation in cells containing high proportion of PUFAs submitted to MB-PDT. Therefore, the subsequent accumulation of lipid peroxidation products can culminate in the permeabilization of the membrane as shown for synthetic membrane models^32^.

Exogenous lipids supplementation could conceivably be relevant in several contexts in vivo, especially for cells that can extract exogenous lipids directly from the bloodstream^33,34^. Raising serum PUFA levels could provide a means to improve the lethality of existing pro-ferroptotic agents against other models of cancer cells^34^. Herein, we have shown that exogenous supplementation of ArA was sufficient to bypass the protective effect conferred by the lack of ACSL4 in MCF-7 cells after MB-PDT. These results pointed PUFAs as key modulators behind the MB-PDT cytotoxicity. Therefore, we showed that the administration of exogenous PUFAs before the photo-oxidation could potentially improve the power of this therapy by tackling tumor cell death resistance to MB-PDT.

In physiological conditions, lipid peroxides are reduced to non-toxic lipid alcohols by GPX4^35^. However, this enzyme is absent or inactive during ferroptosis, resulting in toxic lipid accumulation^36^. We observed that despite GPX4 depletion, not all cell lines underwent ferroptosis after MB-PDT treatment. This effect is consistent with the fact that sensitivity to GPX4 inhibitors varies greatly across cell lines^37^. Our results have further shown that non-tumorigenic BCs presented the highest abundance of CoQ_10_ and were able to survive, despite undergoing lipid peroxidation and GPX4 depletion. Interestingly, CoQ_10_ can trap lipophilic radicals and halt the propagation of lipid peroxidation and prevent ferroptosis^38,39^. Therefore, one can speculate that the GPX4-independent regulation axis of ferroptosis could be operating to suppress phospholipid peroxidation and ferroptosis after photo-oxidative damage in the non-tumorigenic BCs. One can note that this particular effect could be associated with the low side effects of this therapy.

Moreover, we have previously demonstrated the importance of exhaustion of cell antioxidant responses to circumvent resistance to MB-PDT^14^. We now observed that the basal levels of a set of key antioxidant proteins were lower in TNBC cells, further extending the knowledge that these cells exhibit significant metabolic alterations compared to luminal tumors^19,40^. Our data indicated that breast cancer cells were not able to mount such an efficient antioxidant response against photo-oxidation when compared to the one displayed by the non-tumorigenic BCs.

In the present study we also identified that upon photo-oxidation MB induces LMP and disrupts lysosome integrity. Following LMP, a set of RCD can be activated^41–43^. We have earlier shown that apoptosis was not the main cell death mechanism activated in BC submitted to MB-PDT^19^. In line with this, recent reports have pointed that LDCD does not necessarily manifest itself with an apoptotic morphotype and an intriguing connection is emerging between LMP, the adaptive responses to stress, and other regulated necrosis subroutines. For example, lysosomes have an essential role in autophagy and cellular iron homeostasis, being a major source of free iron due to the degradation of ferritin in a process called ferritinophagy^44–46^. Thus, lysosomal damage could increase iron bioavailability to peroxidation reactions. Therefore, upon lipid peroxidation and in the absence of a proper detoxifying response, as occurred in breast tumorigenic cells upon MB-PDT, LMP may facilitate catalysis of iron-dependent reactions and increase ferroptosis susceptibility.

As ferroptosis only occurred in TNBC cells, and that its inhibition did not completely rescue cells from death, we hypothesized that MB-PDT was activating more than one RCD pathway simultaneously. Intriguingly, only tumorigenic cells displayed necroptosis upon MB-PDT. It has been shown that MLKL engagement to undergo necroptosis requires specific phosphatidylinositol phosphates lipids at the plasmatic membrane^47–49^. Our study revealed that non-tumorigenic BC presented lower levels of overall PI, compared to the tumorigenic cells. Therefore, it is reasonable to suppose that lipid composition of normal BCs does not sustain the necroptosis membrane pore formation, also contributing to their resistance to photo-oxidation-induced cell death.

It has previously been shown that necroptosis key players can be degraded in lysosomes, and that inhibition of lysosomal function by LMP leads to these protein accumulation and necroptosis induction^50,51^, strengthening the possibility of a link between LMP and necroptosis. We have shown that, in breast cancer cells, lysosomal cathepsin inhibition suppressed MB-PDT-induced cell death and pMLKL, providing a clear evidence of the existence of cross-talks between LDCD and necroptosis. In this study, LMP appeared as the common event, which was then accompanied by an antioxidant response (in non-tumorigenic cells), necroptosis (in non-invasive tumor cells), or both necroptosis and ferroptosis (in highly aggressive tumor cells). In sum, we provided further molecular mechanisms explaining why BCs displaying distinct molecular makeups are able to undergo different RCD pathways upon the same trigger.

Collectively, our data have provided molecular mechanisms behind a hitherto unexplored therapeutic approach, which have simultaneously activated alternative tumor RCD pathways, while preserving the integrity of most of the non-tumorigenic cells. This fact is of fundamental importance, as despite all recent technological improvements, breast cancer still has significant impact on global health, being disease recurrence and metastasis the bottleneck for an effective clinical treatment^2,3^.

This study contributes to a better understanding of breast cancer susceptibility to photo-oxidation-induced damage. Furthermore, our results could provide the rational and know-how needed to maximize the therapeutic clinical application gain of MB-PDT. Our results support that MB-PDT could be used as a useful adjunct to surgery, to eliminate microscopic residual malignant cells in the post-surgical tumor bed and prevent local and metastatic recurrence without affecting the normal tissue. This strategy gain more importance in the context of TNBC, as metastatic recurrence is the major cause of lethality in patients with this disease.

## Materials and methods

### Cell cultures

MCF-10A, MCF-7, MDA-MB-231 cells (ATCC: CRL-10317™, ATCC HTB-22™, and HTB-26™), MC-38 (RRID:CVCL_B288), and TC-1 (RRID:CVCL_4699) cells were cultured following cell line collection recommendations^19,52^. All cultures were maintained at 37 °C under water-saturated atmosphere containing 5% CO_2_. The authentication of the cell lines used in this work were performed by examining up to 22 polymorphic loci for human and cell line short-tandem-repeat (STR) profiling in accordance with the standard ASN-0002-2011. All cell lines were periodically submitted to PCR and Hoechst staining, to analyze mycoplasma contamination, and were only used in case of negative results.

### Photodynamic treatment (MB-PDT and Hyp-PDT)

Cells were incubated with 20 μM of MB (Labsynth Products, São Paulo, Brazil) or 100 nM of Hypericin for 2 and 16 h, respectively, and then irradiated with a light-emitting diode array (Hypericin: 550 nm; MB: 640 nm). The irradiation time was 16 min with a total light dose was 4.5 J cm^−2^ (fluence rate of 4.7 mW cm^−2^) for both PSs. Inhibitors were incubated with PS solution 2 h before irradiation: Fer-1 (Cayman Chemical, Ann Arbor, Michigan, USA, 1 µM), Nec-1 (Sigma-Aldrich, 10 µM), NSA (Abcam, Cambridge, UK, 5 µM), or CA-074 (Millipore, Burlington, Massachusetts, USA, 400 nM). ArA pretreatment (Sigma-Aldrich, 24 µM) was performed 16 h before MB incubation.

### Cell death evaluation

The total number of cells were determined by counting the nuclei stained with Hoechst 33342 (Sigma-Aldrich) and the number of dead cells determined by the number of nuclei stained with propidium iodide (Sigma-Aldrich). Results were expressed as percentage of dead cell as previously described^19^. A minimum of 500 cells was counted in each experimental condition by two persons where one of them was unaware of the cell type and/or treatment performed. Results were expressed as percentage of dead cells. Each experiment was performed in duplicate. At least three independent experiments were performed for each cell type and condition.

### Transient oligonucleotide transfection

Small interfering RNAs (siRNAs) were transfected into cells using Lipofectamine RNAiMAX (Life Technologies, Carlsbad, CA, USA) according to the manufacturer’s instructions. The Silencer^®^ Select pre-designed siRNA (Life Technologies) for human RIPK3 (5′-GGCAAGUCUGGAUAACGAAtt-3′) or for human MLKL (5′-CCCGUUUCAAGGUGAAGAAtt-3′) were used. “AllStars negative control siRNA” (Qiagen, Venlo, Netherlands) was used as a negative siRNA control of scrambled sequence (siControl). Cells were maintained in culture for a 24 h recovery period before experiments were carried out. The efficiency of transfection/silencing was validated by western blotting.

### Western blottings

Cells were lysed in RIPA Buffer containing protease (Roche, Basel, Switzerland) and phosphatase (Sigma-Aldrich) inhibitor cocktails. Proteins were separated by SDS-polyacrylamide gel electrophoresis and electro transferred onto polyvinylidene difluoride membranes that were subsequently blocked in 5% milk for 1 h room temperature (RT). Primary antibodies (Supplementary Table 1) were diluted in a solution of 5% bovine serum albumin (BSA) in phosphate-buffered saline (PBS) and incubated overnight at 4 °C. Membranes were washed and then incubated at RT for 1 h with horseradish peroxidase-labeled secondary antibodies diluted in a solution of 1% BSA in PBS. Proteins were detected using enhanced chemiluminescence (Millipore Corporation, Billerica, MA, USA). Images were acquired using Uvitec Image System (Cleaver Scientific Limited, Cambridge, UK). Quantitative densitometry was carried out using the ImageJ software (National Institutes of Health). The volume density of the chemiluminescent bands was calculated as integrated optical density × mm^2^ using ImageJ Fiji as previously described^19^.

### Enzyme activities assays

Cells were washed with phosphate buffer (PBSA: NaCl 137 mM, KCl 2.7 mM, Na_2_HPO_4_ 10 mM, KH_2_PO_4_ 1.8 mM pH 7.2) and detached from the plate using trypsin solution (0.5% p/v). The cells were then centrifuged at 800 × *g* for 2 min. Cell pellets were washed with PBSA and resuspended in 2 mL PBSA. Samples were then homogenized and centrifuged at 4 °C, 700 × *g* for 10 min. The supernatants were collected and centrifuged at 4 °C, 25,000 × *g* for 2 h for cytosol and organelles fractionation. Cytosolic fractions were used in cathepsin B/L kinetics assays using Z-FR-MCA as substrate (10 µM) in 100 mM citrate phosphate buffer pH 6. Protease activity was evaluated at an excitation wavelength of 380 nm and an emission wavelength of 460 nm using a 96-well plate in a spectrofluorometer (SpectraMAX M2, Molecular Devices, Sunnyvale, CA, USA). Fluorescence intensity values were collected every 5 min intervals for 1 h. Activity units were calculated as: [relative fluorescence units/min]/number of cells. The determination of G6PD activity was performed as already described^53^. Each experiment was performed in duplicate. At least three independent experiments were performed for each cell type and condition.

### Lipid peroxidation analysis

Lipid peroxidation was detected and quantified using BODIPY-C11 probe by fluorescent microscopy. Images were quantified using ImageJ Fiji. Cells were imaged using a fully motorized Leica DMi8 widefield microscope (from Leica Microsystems) using the fluorescein isothiocyanate and Texas Red filter sets, and a ×20 objective. All imaging acquisition parameters were kept constant for each experiment. Images were quantified using ImageJ Fiji. Cell outlines were free-handed drawn on the bright-field channel to generate a cell selection mask for quantifying the fluorescence intensity in the green and red channels. Oxidation of BODIPY-C11 581/591 was calculated as the ratio of the green (fluorescence emission of the oxidized probe)/red fluorescence mean intensity (fluorescence emission of reduced probe) within the cell outlines. Imaging was performed on two independent biological replicates. In each independent experiment at least 4 different images (100 cells) per condition were analyzed. Each experiment was performed in duplicate. At least three independent experiments were performed for each cell type and condition.

### Lipidomic analysis

Non-targeted lipidomic analysis of major lipids was performed by reversed-phase ultra-high-performance liquid chromatography coupled to electrospray ionization time-of-flight mass spectrometry as previously described^54^. The lipid quantification was performed with MultiQuant^®^, in which peak areas of precursor ions were normalized to those of the internal standards. Final data were expressed as mass of lipid species per mass of total proteins, determined by BCA Protein Assay Kit (Thermo) following the manufacturer’s instructions. Lipids were annotated according to their lipid subclass. Individual lipids were also grouped as the total number of double bonds in saturated (no double bounds), monounsaturated (presence of one double bound), or polyunsaturated (presence of more than one double bound) (Supplementary Table 2).

### LIP measurement

LIP was given as sum of the concentrations of iron ([Fe]) and calcein-bound Fe ([CA-Fe]), normalized to the total intracellular calcein ([CA]t), whereby LIPN = LIP/[CA]t. We followed the rationale for fluorescence determination of LIP developed by Epsztejn and collaborators^54^ with minor modifications. The [CA-Fe] was obtained from the relationship [CA-Fe] = ΔF*[CA]t. [Fe] was calculated from CA-Fe dissociation constant: *Kd* = [CA]t*[Fe]/[CA-Fe]), using the experimental values of [CA-Fe] and [CA]*t*, and the *Kd* in cells value of (0.22) obtained from the original paper^55^. CA, CA-AM, and SIH were generous gifts from Dr. Breno Pannia Espósito, Chemistry Institute of the University of São Paulo, Brazil. Each experiment was performed in duplicate. At least three independent experiments were performed for each cell type and condition.

### GSH measurement

Cells were seeded in cell culture-treated dishes (100 mm) at initial density of 2.6 × 10^6^ cells. One hour post MB-PDT, the cells were washed with phosphate buffer (PBSA: NaCl 137 mM, KCl 2.7 mM, Na_2_HPO_4_ 10 mM, KH_2_PO_4_ 1.8 mM pH 7.2), removed from the plates by scrapping with 100 µL of PBSA. GSH was measured after 1 h of MB-PDT and analyzed using a fluorimetric detection assay (ab138881, Abcam, UK) according to the manufacturer’s instructions. GSH concentration was calculated by interpolation of a standard curve and results were expressed as: [nmol/mg of total protein]. Each experiment was performed in duplicate. At least three independent experiments were performed for each cell type and condition.

### Statistical analysis

All results were analyzed for Gaussian distribution and passed the normality test (the number of independent experiments was chosen to present a normal distribution). The statistical differences between group means were tested by one-way analysis of variance (ANOVA) followed by Tukey’s post test for multiple comparisons or by two-way ANOVA followed by Bonferroni’s post test for multiple comparisons. The variances of the data meet the criteria for application of the different post tests used. For PCA in lipidomic studies, statistical analysis was performed with MetaboAnalyst website. A value of *p* < 0.05 was considered as statistically significant in all analysis.

All data presented in this manuscript are available upon request to the authors.

## Supplementary information

Supplementary table 2

Supplementary table 2

Supplementary figure Legends

Supplemamentary figure 1

Supplementary Figure 2

Supplementary figure 3

Supplementary figure 4

Supplementary figure 5

Supplementary figure 6
