# Supplementary material for: Distinct photo-oxidation-induced cell death pathways lead to selective killing of human breast cancer cells
Source: Cell Death Dis. 2020 Dec 14;11(12):1070. doi: 10.1038/s41419-020-03275-2 (PMC7736888; doi:10.1038/s41419-020-03275-2)
Supplement: Supplementary file 1 — Supplementary table 2 [file 41419_2020_3275_MOESM1_ESM.docx]

Supplementary Table 1: List of Antibodies.

| *Protein* | *Company* | *Catalog* | *Dilution* |
| --- | --- | --- | --- |
| RIPK1 | BD | #610458 | 1:1000 |
| RIPK3 | Cell Signaling | #13526 | 1:1000 |
| phospho(S345) MLKL | abcam | ab196436 | 1:1000 |
| MLKL | abcam | ab184718 | 1:1000 |
| ACSL4 | Santa Cruz | sc-271800 | 1:200 |
| GPX4 | abcam | ab125066 | 1:1000 |
| G6PD | abcam | ab993 | 1:2000 |
| NRF2 | abcam | ab137550 | 1:1000 |
| SOD1 | abcam | ab51254 | 1:2000 |
| SOD2 | abcam | ab16956 | 1:1000 |
| Glutathione Reductase | abcam | ab128933 | 1:2000 |
| Glutathione Synthetase | abcam | ab133592 | 1:2000 |
| alpha-tubulin clone B-5-1-2 | Sigma-Aldrich | T5168 | 1:10.000 |
| anti-rabbit | Vector Laboratories | PI1000 | 1:1000 |
| anti-mouse | Vector Laboratories | PI2000 | 1:1000 |
