# Supplementary material for: Distinct photo-oxidation-induced cell death pathways lead to selective killing of human breast cancer cells
Source: Cell Death Dis. 2020 Dec 14;11(12):1070. doi: 10.1038/s41419-020-03275-2 (PMC7736888; doi:10.1038/s41419-020-03275-2)
Supplement: Supplementary file 2 — Supplementary table 2 [file 41419_2020_3275_MOESM2_ESM.docx]

Supplementary Table 2: Lipid internal standards (from Avanti Polar Lipids Inc., Alabaster, Alabama, USA)

| *Internal standards* | *Lipid* | *Concentration (ng/μL)* |
| --- | --- | --- |
| cholest-5-en-3ß-yl (decanoate) | CE 10:0 | 10 |
| N-decanoyl-D-erytro-sphingosine | Cer d18:1/10:0 | 10 |
| N-heptadecanoyl-D-erythro-sphingosine | Cer d18:1/17:0 | 10 |
| 1′,3′-bis[1,2-dimyristoyl-sn-glycero-3-phospho]-glycerol | CL 14:0 x 4 | 10 |
| 1-heptadecanoyl-2-hydroxy-sn-glycero-3-phosphocholine | LPC 17:0 | 10 |
| 1-(10Z-heptadecenoyl)-sn-glycero-3-phosphoethanolamine | LPE 17:1 | 10 |
| 1,2-diheptadecanoyl-sn-glycero-3-phosphate | PA 17:0/17:0 | 10 |
| 1,2-dimyristoyl-sn-glycero-3-phosphocholine | PC 14:0/14:0 | 10 |
| 1,2-diheptadecanoyl-sn-glycero-3-phosphocholine | PC 17:0/17:0 | 10 |
| 1,2-dimyristoyl-sn-glycero-3-phosphoethanolamine | PE 14:0/14:0 | 10 |
| 1,2-diheptadecanoyl-sn-glycero-3-phosphoethanolamine | PE 17:0/17:0 | 10 |
| 1,2-diheptadecanoyl-sn-glycero-3-phospho-(1'-rac-glycerol) | PG 17:0/17:0 | 10 |
| 1,2-diheptadecanoyl-sn-glycero-3-phospho-L-serine | PS 17:0/17:0 | 10 |
| N-heptadecanoyl-D-erythro-sphingosylphosphorylcholine | SG d18:1/17:0 | 10 |
| 1,2,3-tritetradecanoyl-sn-glycerol | TG 14:0 x 3 | 10 |
| 1,2,3-triheptadecanoyl-sn-glycerol | TG 17:0 x 3 | 10 |
