# Supplementary material for: Distinct photo-oxidation-induced cell death pathways lead to selective killing of human breast cancer cells
Source: Cell Death Dis. 2020 Dec 14;11(12):1070. doi: 10.1038/s41419-020-03275-2 (PMC7736888; doi:10.1038/s41419-020-03275-2)
Supplement: Supplementary file 3 — Supplementary figure Legends [file 41419_2020_3275_MOESM3_ESM.docx]

**Supplementary Material:**

**Supplementary Figure Legends**

**Supplementary Figure 1: Basal lipid composition of breast cells.** Lipids were extracted from the three cell types. (A) Neutral lipid abundance: Cholesteryl Esters (CE), Ceramides (Cer), Diacylglycerides (DG) and Triacylglycerides (TG). (B) Abundance of phosphatidylcholine (PC), plasmanyl (o)- and plasmenyl (p)-PCs (oPC and pPC respectively). (C) Abundance of Adrenic Acid (AdrA)-containing lipids. (D) Abundance of Arachidonic Acid (ArA) esterified in phosphatidyletanolamine (PE). (E) Abundance of AdrA esterified in PE. *** p<0.001; ** p<0.005; *p<0.05 *vs* MCF-10A. Results are presented as mean ± S.E.M. n=3 independent experiments. Dot colors representation: MCF-10A in red; MCF-7 in green; MDA-MB-231 in blue.

**Supplementary Figure 2: Basal abundance of ferroptosis key players.** (A) Representative pictures of Western blots and the corresponding quantification of (B) ACSL4 and (C) GPX4 **** p<0.0001; *** p<0.001 *vs* MCF-10A. (D) GSH levels in breast cells after 1h of MB-PDT treatment. **** p<0.0001; ** p<0.005 *vs* control of respective cell line. Results are presented as mean ± S.E.M. n=3 independent experiments. Dot colors representation: MCF-10A in red; MCF-7 in green; MDA-MB-231 in blue.

**Supplementary Figure 3: Basal abundance of antioxidant-related proteins.** (A) Representative pictures of Western blots and the corresponding quantification of (B) G6PD, (C) NRF2, (D) SOD1, (E) SOD2, (F) GR- glutathione reductase and (G) GS-glutathione synthetase. ***: p<0.001; **: p<0.005; *: p<0.05 *vs* MCF-10A. Results are presented as mean ± S.E.M. n≥3 independent experiments. Dot colors representation: MCF-10A in red; MCF-7 in green; MDA-MB-231 in blue.

**Supplementary Figure 4: MB-PDT induces necroptosis in breast tumor cells.** (A) Representative pictures of Western blots and the corresponding quantification of basal levels of (B) RIPK1, (C) RIPK3, (D) MLKL.

(E, F) RIPK3 or (G, H) MLKL was silenced or not in the cells, which were then submitted to MB-PDT. Cells were submitted or not to MB-PDT and cell death was analysed after 1, 3 or 24h of cell treatment. Right panels: representative Western blot analysis of RIPK3 and MLKL protein levels. ****: p<0.0001; ***: p<0.001; **: p<0.005; *: p<0.05 *vs* MCF-10A. Results are presented as mean ± S.E.M. n≥3 independent experiments.

**Supplementary Figure 5: MB-PDT induces necroptosis in lung tumor cells and apoptosis in colon tumor cells.** (A) Cell death induction after 1, 3,6 or 24 h of MB-PDT treatment in TC-1 lung tumor cells wild type (TC-1_WT) or knockout for RIPK3 or MLKL (TC-1_RIPK3^-/-^ and _MLKL^-/-^ respectively) and MC-38 colon tumor cells. *: p<0.05 *vs* TC-1_WT. Results are presented as mean ± S.E.M. n≥3 independent experiments. (B) Percentage of cell death after MB-PDT (6 or 24h) in MC-38 cells pretreated or not with z-VAD-FMK (Z-VAD, 20 µM) or Necrostatin-1s (Nec-1s, 10 µM). *: p<0.05; ****: p<0.0001 vs. corresponding MB-PDT. Results are presented as mean ± S.E.M. n≥3 independent experiments (C) Percentage of cell death after MB-PDT (6 or 24h) in TC-1 wild type cells pretreated or not with z-VAD-FMK (Z-VAD, 20 µM), Necrostatin-1s (Nec-1s, 10 µM) or Ferrostatin-1 (Fer-1, 1 µM). *: p<0.05; ***: p<0.001 vs. corresponding MB-PDT. Results are presented as mean ± S.E.M. n≥3 independent experiments (D) Graphical abstract of the results representing the mechanisms activated by MB-PDT in TC-1 or MC-38 cells as indicated, indicating that MB-PDT was able to activate necroptosis or apoptosis depending on the cell type.

**Supplementary Figure 6: Hyp-PDT induces apoptosis in breast tumor cells and necrosis in non-tumorigenic cells.** (A) Percentage of cell death after 1, 3 or 24 h of Hypericin-PDT (Hyp-PDT) treatment in breast cells. ****: p<0.0001; **: p<0.005 *vs* MCF-10A. Results are presented as mean ± S.E.M. n≥3 independent experiments. (B) Representative images of breast cells stained with Hoechst 33342 (blue; total nuclei) and PI (red; cells bearing permeabilized membranes) after 24h of being submitted or not to Hyp-PDT. (C, D, E) Death percentage in (C) MCF-7, (D) MCF-10A and (E) MDA-MB-231 cells after 3 or 24h of being pretreated in the presence or in the absence of z-VAD-FMK (Z-VAD, 20 µM), Necrosulfonamide (NSA, 10 µM), Ferrostatin-1 (Fer-1, 1 µM) or CA-074 (5 µM) and the submitted or not to Hyp-PDT. **: p<0.005 *vs* corresponding Hyp-PDT. Results are presented as mean ± S.E.M. n≥3 independent experiments. (F) Graphical abstract of the above results representing the cell death mechanisms activated by Hyp-PDT in breast cells indicating that MCF-7 cancer cells were resistant to Hyp-PDT treatment while apoptosis and necrosis were triggered in MDA-MB-231 cancer cells and MCF-10A non-tumorigenic cells respectively.
