# Supplementary figures and images for: Distinct photo-oxidation-induced cell death pathways lead to selective killing of human breast cancer cells
Source: Cell Death Dis. 2020 Dec 14;11(12):1070. doi: 10.1038/s41419-020-03275-2 (PMC7736888; doi:10.1038/s41419-020-03275-2)

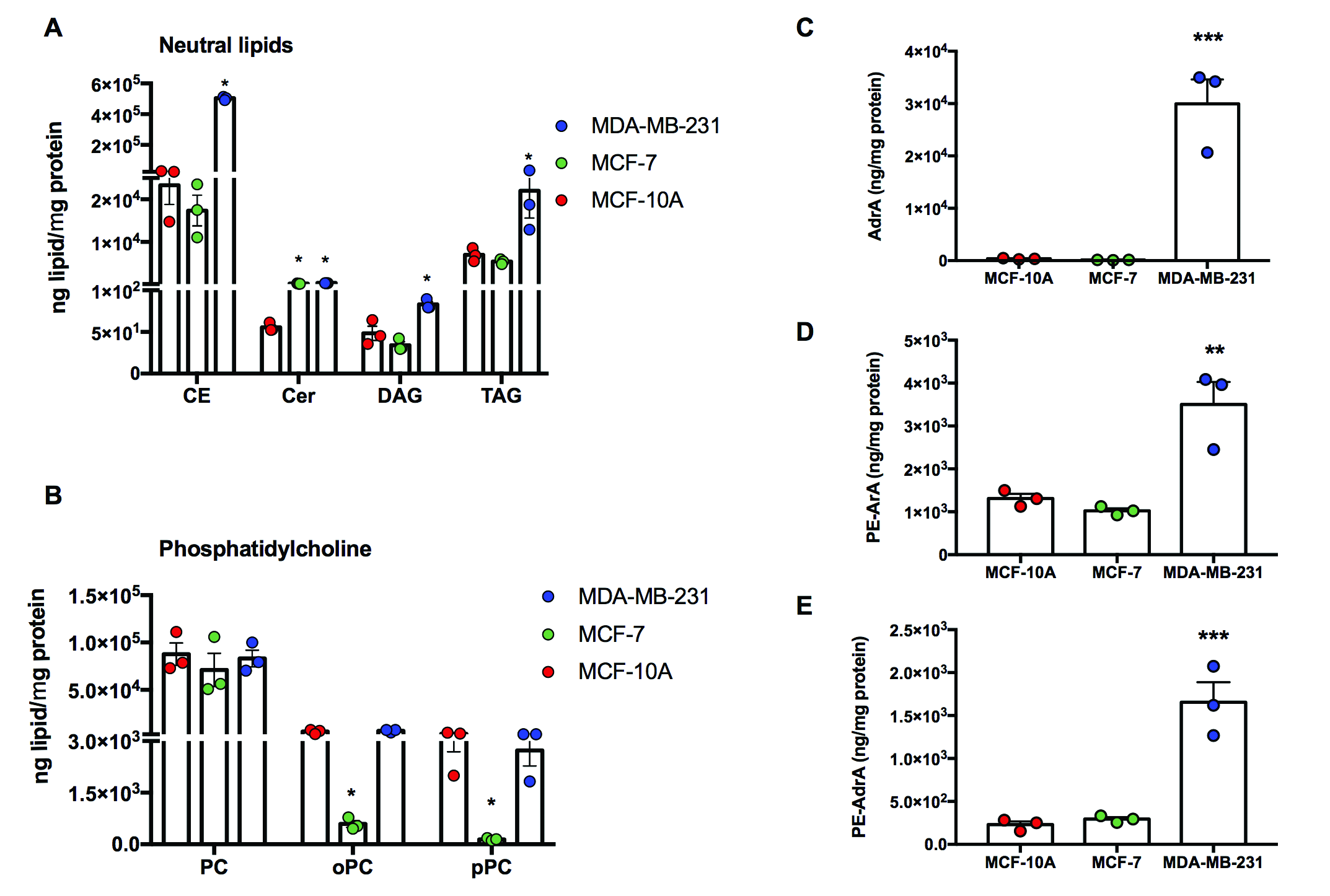

Supplement: Supplementary file 4 — Supplemamentary figure 1 [file 41419_2020_3275_MOESM4_ESM.tif]

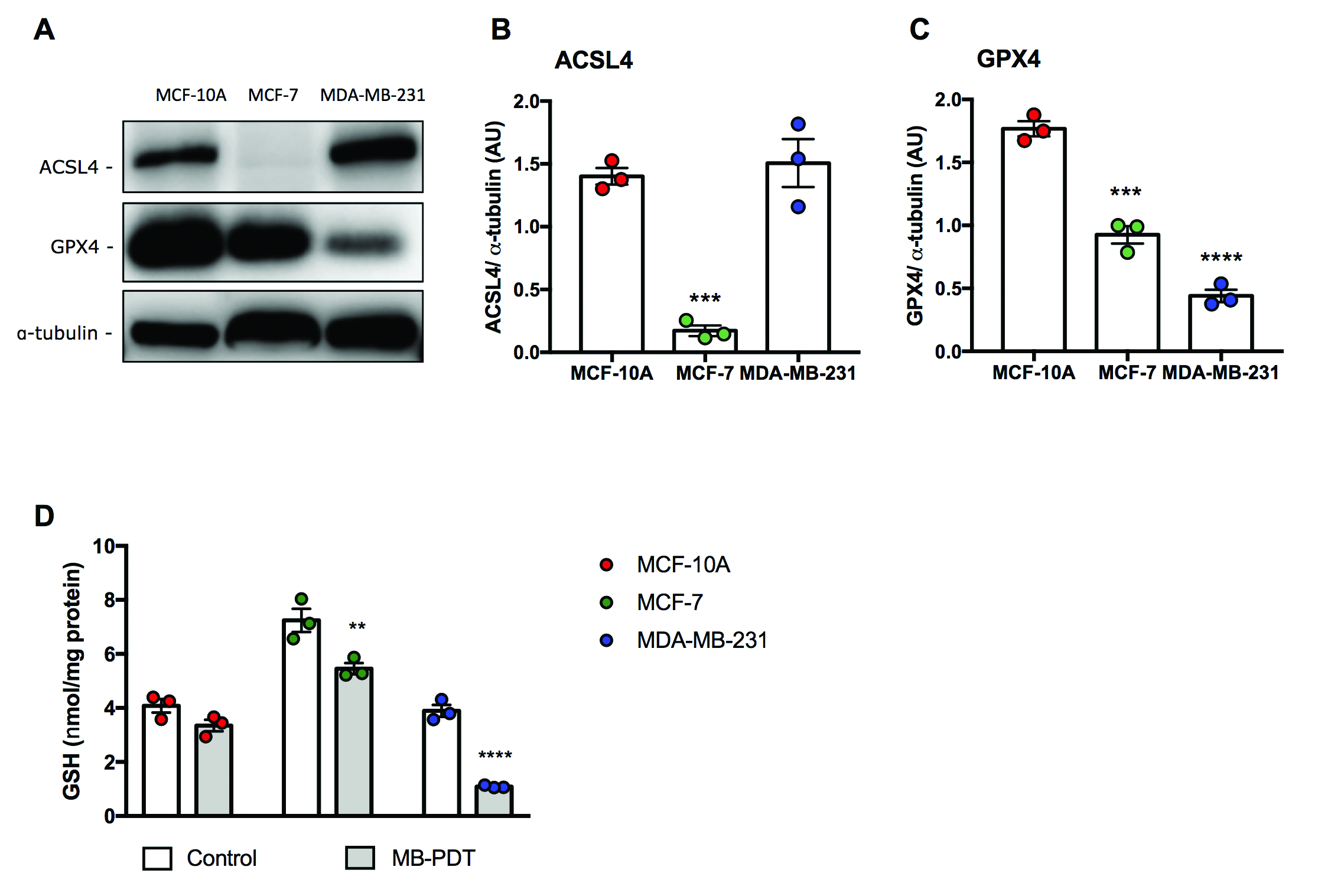

Supplement: Supplementary file 5 — Supplementary Figure 2 [file 41419_2020_3275_MOESM5_ESM.tif]

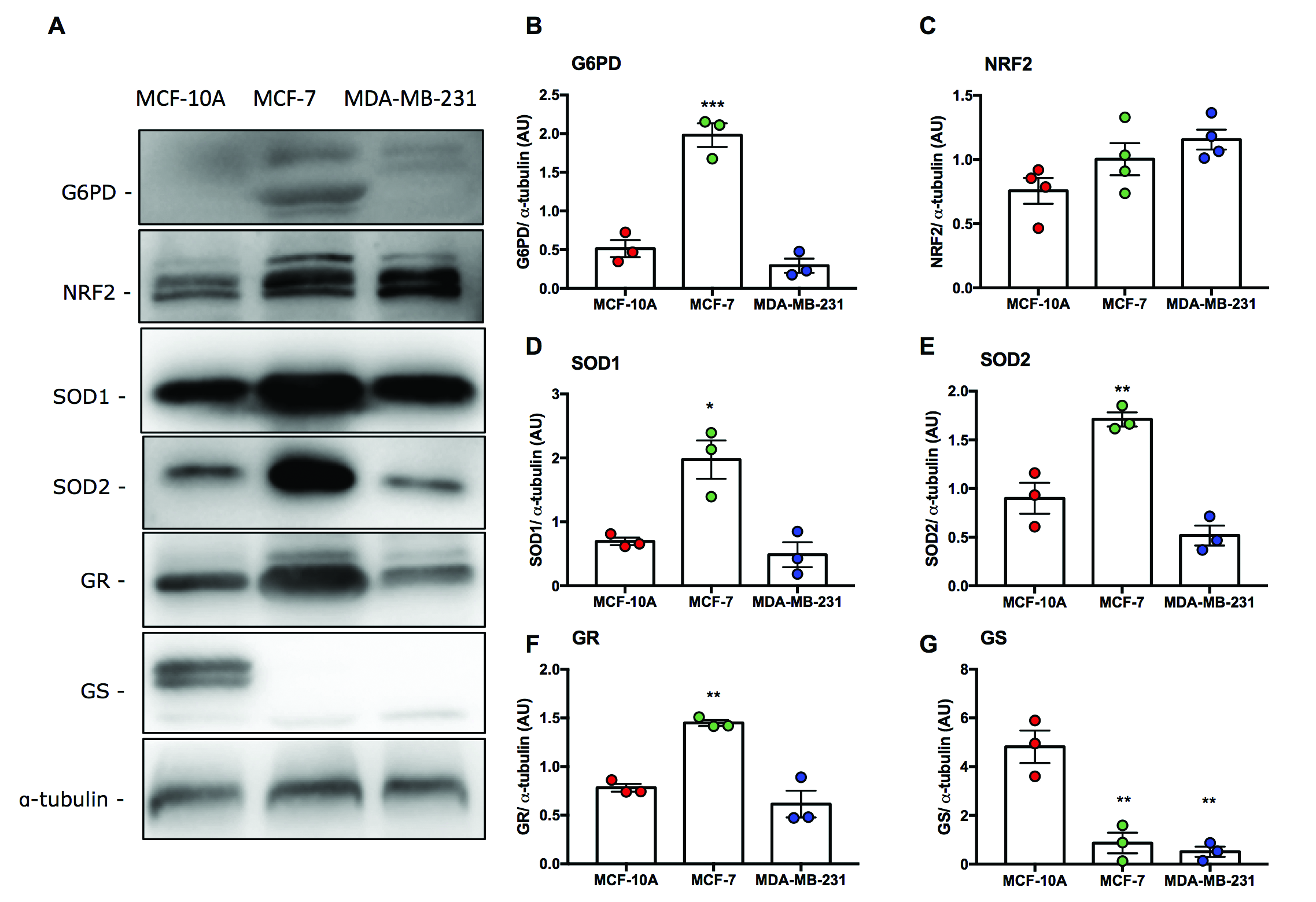

Supplement: Supplementary file 6 — Supplementary figure 3 [file 41419_2020_3275_MOESM6_ESM.tif]

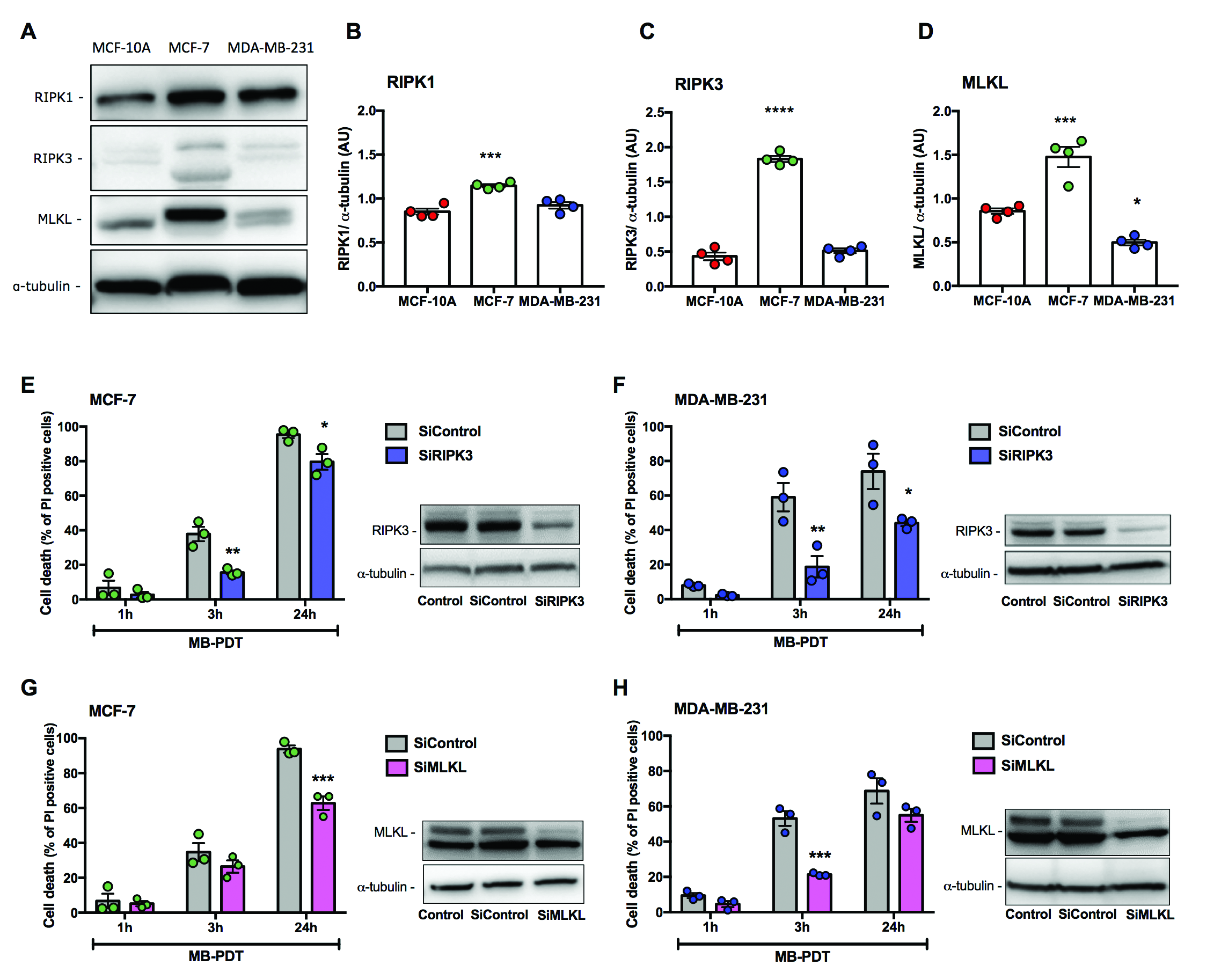

Supplement: Supplementary file 7 — Supplementary figure 4 [file 41419_2020_3275_MOESM7_ESM.tif]

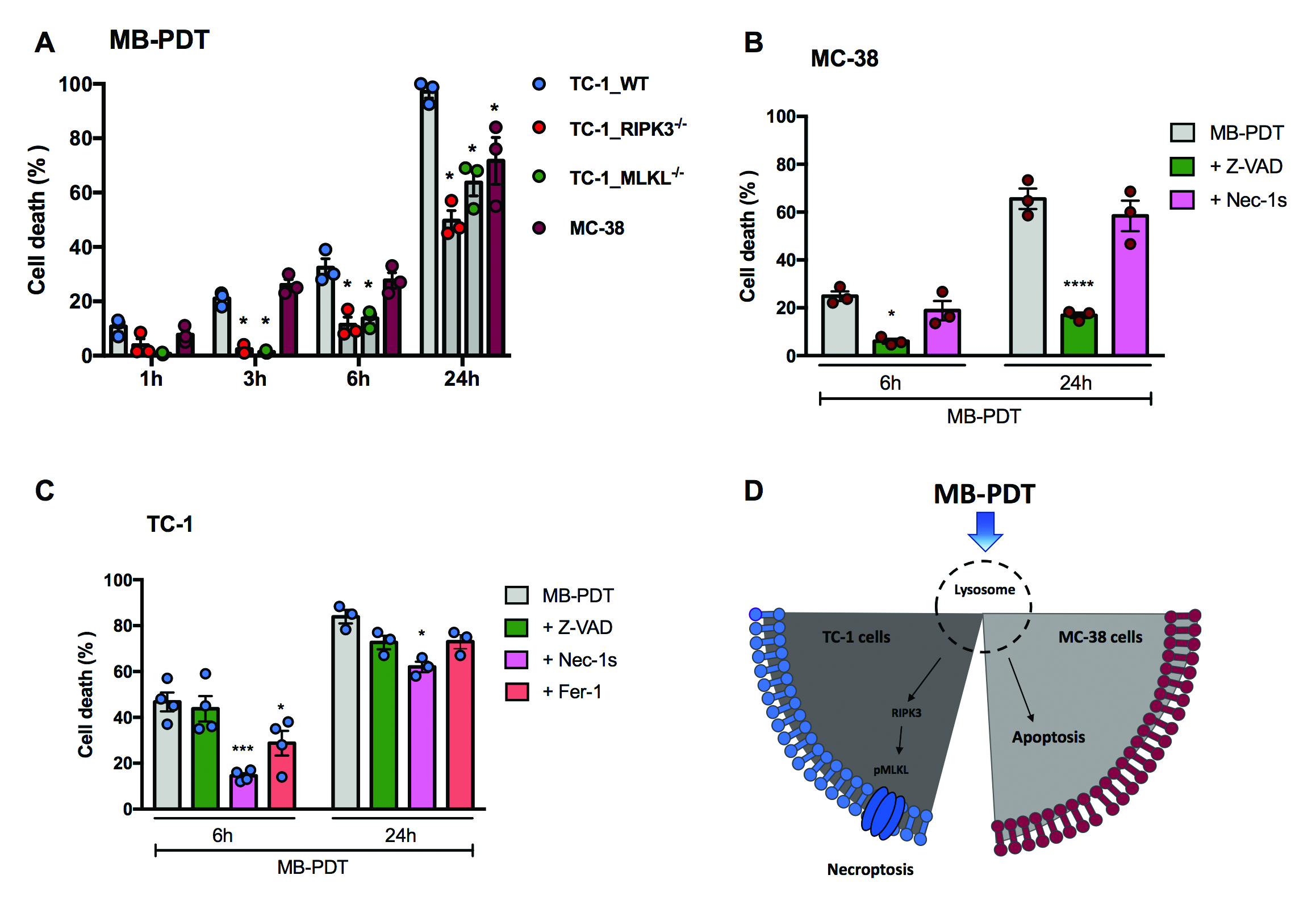

Supplement: Supplementary file 8 — Supplementary figure 5 [file 41419_2020_3275_MOESM8_ESM.tif]

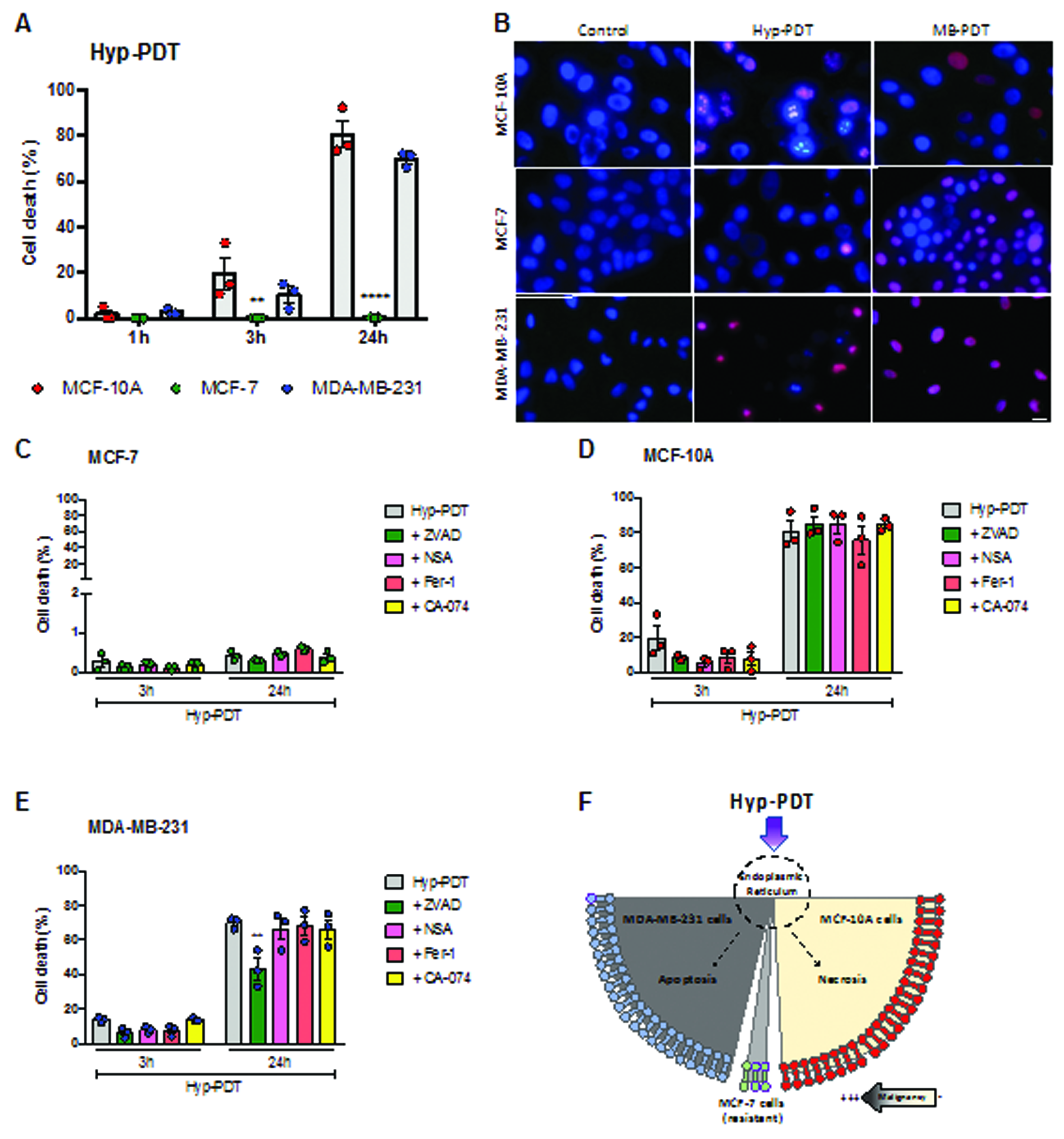

Supplement: Supplementary file 9 — Supplementary figure 6 [file 41419_2020_3275_MOESM9_ESM.tif]
